# Mr. Skrimshire, on Scarlet Fever

**Published:** 1801-11

**Authors:** F. Skrimshire

**Affiliations:** Kettering, Northamptonshire


### *To the Editors of the Medical and Phyſical Journal.*

GENTLEMEN,

THE *Scarlatina Anginosa*, in a form ſimilar to the epidemic deſcribed by Dr. Withering, has been very prevalent this ſummer and autumn in ſome of the neighbouring towns. The infection was brought into one family of this place, but fortunately ſpread no further. In this family four of the children were attacked with it. The fever in none of them ran very high; the throat was but ſlightly affected; and only in one caſe was there delirium. In one other caſe there was none of the ſcarlet eruption; but in all, the febrile ſymptoms were ſucceeded in a few days by thoſe of dropſy, which commenced with general anaſarca. To this ſucceeded aſcites, and then hydrothorax, and three of the caſes terminated fatally, within a month of the febrile attack. In the progreſs of the dropſy numerous petechiæ appeared in all the four caſes, chiefly on the trunk and upper extremities. In the three unfortunate caſes they made their appearance only a few days previous to their death, and they were very numerous on the fourth, at the time I was called upon to viſit the family. I made my firſt viſit the day after that on which the ſecond child died, and found the mother hourly expecting the death of the third, and in great anxiety about the fourth. I ordered Port wine for both, an alkaline ſolution for the third, and a gentle emetic for the fourth. They both ſeemed ſomewhat relieved next day, and I deſired the alkaline ſolution to be given to both, and ordered an electuary of jalap and cream of tartar to be taken freely.

By

By this plan the fwellings were confiderably reduced, but the dyfpnœa, and other fymptoms of water in the cheft, feemed to increafe in the third child, and he died two days after. I had the fatisfaction of examining this patient after death, and found about four ounces of water in the pericardium, and the fame quantity in the cavity of the thorax. There were feven or eight ounces in the abdomen, and none in the ventricles of the brain. The liver feemed fomewhat enlarged, but otherwife healthy, the lungs were perfectly found, nor were there any other morbid appearances. The liquor that I procured from the cavity of the abdomen for examination, was partly coagulated in a few minutes by mere expofure to the air. The coagulum, which was about one half of the whole, was not diffolved, but corrugated, when hot water was poured upon it, and it was infoluble in cold aqua ammon. pur. It muft therefore have been the fibrine of the blood. That part of the liquor which remained fluid was not coagulable by a heat even greater than 160, at which temperature the ferum of the blood coagulates, nor was it coagulable by alcohol, but it was fo by Goulard's extract. This therefore was the *serosity* of the blood, containing no albumen; and the dropfical water, in this cafe, was the blood deprived of its colouring particles, and its albumen.

The other child, which had lefs dyfpnœa, though confiderable fwelling of the abdomen, and general anafarca, and likewife numerous petechiæ, gradually recovered by the ufe of the alkaline folution, the purging electuary, and latterly the tincture of digitalis. Care was taken to fupport her ftrength by a nourifhing diet and Port wine during the operation of thefe medicines. It is right to obferve that this family had fared very hardly during laft winter, and had juft begun to experience the benefits of this year's abundant harveft. The family from whom thefe children took the difeafe, loft one child from the fame dropfical fymptoms accompanied with petechiæ, and a fecond was faved after thefe fymptoms had appeared, by the ufe (if I am rightly informed,) of brifk cathartics and tincture of digitalis.

The poor of this parifh, where the late fcarcity has been very particularly felt, have fuffered confiderably from typhus fever, which has been often fatal, and I think more frequently than ufual, attended with petechiæ.

If the above obfervations are thought worthy of being communicated to the public, they are much at your fervice.

*Kettering, Northamptonshire,*
*October* 8, 1801.

I am, &c.
F. SKRIMSHIRE.

